# Intelligent UAV Deployment for a Disaster-Resilient Wireless Network

**DOI:** 10.3390/s20216140

**Published:** 2020-10-28

**Authors:** Hassaan Hydher, Dushantha Nalin K. Jayakody, Kasun T. Hemachandra, Tharaka Samarasinghe

**Affiliations:** 1 Centre for Telecommunication Research, School of Engineering, Sri Lanka Technological Campus, Ingiriya Road, Padukka 10500, Colombo, Sri Lanka; hassaanh@sltc.ac.lk; 2 Department of Electronic and Telecommunication Engineering, University of Moratuwa, Moratuwa 10400, Sri Lanka; kasunh@uom.lk (K.T.H.); tharakas@uom.lk (T.S.); 3 School of Computer Science and Robotics, National Research Tomsk Polytechnic University, 634050 Tomsk, Russia

**Keywords:** aerial base station, average spectral efficiency, interference mitigation, particle swarm optimization, unmanned aerial vehicles

## Abstract

Deployment of unmanned aerial vehicles (UAVs) as aerial base stations (ABSs) has been considered to be a feasible solution to provide network coverage in scenarios where the conventional terrestrial network is overloaded or inaccessible due to an emergency situation. This article studies the problem of optimal placement of the UAVs as ABSs to enable network connectivity for the users in such a scenario. The main contributions of this work include a less complex approach to optimally position the UAVs and to assign user equipment (UE) to each ABS, such that the total spectral efficiency (TSE) of the network is maximized, while maintaining a minimum QoS requirement for the UEs. The main advantage of the proposed approach is that it only requires the knowledge of UE and ABS locations and statistical channel state information. The optimal 2-dimensional (2D) positions of the ABSs and the UE assignments are found using K-means clustering and a stable marriage approach, considering the characteristics of the air-to-ground propagation channels, the impact of co-channel interference from other ABSs, and the energy constraints of the ABSs. Two approaches are proposed to find the optimal altitudes of the ABSs, using search space constrained exhaustive search and particle swarm optimization (PSO). The numerical results show that the PSO-based approach results in higher TSE compared to the exhaustive search-based approach in dense networks, consuming similar amount of energy for ABS movements. Both approaches lead up to approximately 8-fold energy savings compared to ABS placement using naive exhaustive search.

## 1. Introduction

Unmanned aerial vehicles (UAVs) have numerous applications in fields such as military, disaster management, search and rescue, security, photography, etc. [1]. Continuous improvements in flight time endurance, payload capacity, and advanced control mechanisms have paved the way to applications of UAVs in new fields such as agriculture, transportation, and, most recently, in wireless communications. Three main use cases of UAVs in wireless communications have been identified: A UAV may be used as an aerial base station (ABS), an aerial relay, or an aerial mobile station (MS). Out of the use cases, deploying UAVs as ABSs to enhance the coverage and capacity of terrestrial wireless systems has attracted significant research attention. Potential applications include supplementing the overloaded terrestrial network due to large crowds and providing temporary coverage in areas where the terrestrial network is unavailable due to a natural disaster or an emergency situation [2,3]. This article proposes a scheme to optimally position a set of ABSs to provide network coverage to a group of users, who have lost connectivity to the terrestrial base station (BS) due to a disaster situation.

The high mobility, quick deployment capability, and low capital expenditure of UAV ABSs make them effective solutions in the above scenarios [1]. Furthermore, UAV ABSs increase the probability of line of sight (LoS) links, which enhance the received signal quality compared to non-line of sight (NLoS) links available in terrestrial BS networks. However, the successful deployment of UAVs as ABSs requires overcoming several challenges. The higher mobility demands complex control mechanisms [1] and effective trajectory planning, while on-demand deployment requires self-organizing network (SON) capability [4]. Furthermore, the higher probability of LoS links increases the co-channel interference (CCI). Thus, the control algorithm plays a crucial role in such a network, as it should focus on several aspects that ensure proper functioning of the system. These include radio resource management, interference management, channel estimation and prediction, placement, user association, energy efficiency, and trajectory planning. Therefore, significant research attention has been focused on intelligent planning of UAV networks recently.

The recent works on UAV networks mainly focus on network coverage [5,6,7,8,9], quality of connectivity [10], network topology [11], target tracking [12], and utilizing the UAVs as ABSs to serve the user equipments (UEs). In general, all UAV ABSs are connected to each other and to a central control station [5,6,7,8,10,11,12,13,14,15]. The works in the literature consider both centralized [5,6,16,17,18,19,20] and decentralized approaches [8,21,22,23] for the deployment and control of UAV networks. Centralized algorithms are capable of providing highly accurate decisions compared to the decentralized approaches. However, the requirement of having global information at a central location may lead to significant overhead and delay, depending on the application. In contrast, distributed algorithms share limited information and make intelligent decisions through locally available data, resulting in lower overhead and delay in the system. However, as distributed algorithms completely rely on device intelligence and local information, limitations on device intelligence and inaccurate information may lead to system failure. In our work, we propose two low complexity ABS positioning algorithms and a UE assignment scheme, which deems a centralized approach more feasible.

Energy efficiency is paramount for ABSs as the power supply is restricted. There are several techniques introduced to alleviate this issue such as radio frequency energy harvesting, wireless power transfer [24], simultaneous information and power transfer, and self-interference exploitation [25]. However, it is well known that the energy spent in maneuvering the UAVs dominates the energy efficiency, thus UAV deployment and trajectory optimization are crucial for the success of such a network. There are algorithmic approaches [6] that study the deployment problem, with a focus on increasing the energy efficiency without sacrificing the quality of service (QoS) requirements. To this end, machine learning (ML)-based deployment approaches facilitate comparatively quick responses and lower data overheads. In [26], genetic algorithm (GA) and reinforcement learning are used for optimal deployment and user assignment. Similarly, GA with the hill climbing algorithm (HCA) is used in [27] to enable the communication services and explore the unidentified victims. In [27], initial deployment is done through GA and then the HCA is used to adapt the system to the conditions.

Apart from machine learning algorithms, heuristic algorithms have also been used to solve the UAV placement problem. Although heuristic approaches do not always guarantee the global optimum, can be useful in many applications. Heuristic approaches find an optimal solution without searching over the entire problem space. Therefore, it outperforms typical exhaustive approaches in terms of number of iterations or latency. However, the computational intensity increases exponentially with the number of dimensions of the problem space in heuristic approaches. One example for a heuristic approach is particle swarm optimization (PSO). This optimization approach is originally proposed by J. Kennedy and R. Eberhart in 1995 [28]. The inspiration of this algorithm is the behavior of a bird flock. The PSO-based 3D placement of ABSs is studied in [29]. Although PSO provides superior average performance, the performance for a given instance cannot be guaranteed as it follows a heuristic approach. Therefore, completely relying on PSO may result in severe performance degradation.

A significant contribution is made in [14] with regards to UAV deployment, and this can be considered to be the most related reference to our work. In [14], the authors have used a matching algorithm and a clustering algorithm to find the best 2D position and the UE assignment for a given altitude. Then, a game theoretic approach is used to find the optimal altitudes of the ABSs. Initially, the ABSs are randomly placed in the area of interest, and the ABSs continuously move in the 2D plane until they reach the best 2D position. Subsequently, the altitudes of the ABSs are changed based on the aforementioned game theoretic approach, and the 2D positions and the UE assignments are further fine tuned taking the adjusted altitudes into consideration. This iterative process continues such that the ABSs repetitively change their locations until they reach the optimal positions. A main drawback of this approach is the time and the energy spent in continuously moving the ABSs. It is important to note that the time spent for UAV maneuvering is significantly large compared to the channel coherence time. Therefore, in a practical scenario, the estimated channel state information (CSI) can be less accurate and cause performance degradation. Furthermore, the energy limitations of ABSs have not been taken into account in [14]. There are several other limitations and assumptions found in the literature that do not fully reflect realistic attributes of UAV ABSs. For example, in [26], an interference-free environment is assumed and CCI is neglected. However, due to the higher probability of LoS propagation in the ABS to UE links, CCI from other ABSs is inevitable.

Considering the limitations of the previous work, in this paper we propose a centralized and less complex approach that relies on the average statistics of the channel and eliminates the necessity to continuously move the ABSs. The only information required at the central controller (CC) is the locations of the UEs and the initial locations of the ABSs. With the available location data, we find the optimal locations of the ABSs and the UE assignment for each ABS at the CC, where there is sufficient computational power to provide a rapid solution. According to the decision of the CC, the ABSs can directly change their position from the initial position to the optimal position in one step, making it a quick and energy efficient approach.

The deployment problem is divided into three phases, which are 2D deployment, UE assignment, and the altitude selection. The approach taken for 2D deployment and the UE assignment has similarities to [14] as they stem on a clustering algorithm and a matching algorithm, respectively. The approaches significantly differ in the third step, which is the altitude selection. We propose two methods for altitude selection. The first method performs an exhaustive search among a discrete set of altitude values, without allowing altitude diversity among ABSs. This means all ABSs operate at a common altitude. We effectively truncate the search space by using properties of our objective function. On the other hand, the second method facilitates altitude diversity, and the altitudes of the ABSs are decided through a PSO algorithm. Our proposed algorithms keep track of the available energy in the batteries of the ABSs, and take them into consideration in making the decision on the ABS deployment, making them further different from the schemes proposed in [14].

Our work considers the impact of CCI in the ABS deployment process. Furthermore, we focus on fully utilizing the spatial diversity by deploying omnidirectional antennas. In addition, being different to our work, the impact of LoS is not considered in [9,25,26,30] for small-scale fading, which can significantly affect the performance in dense urban environments. Moreover, the altitude of the UAVs are not taken into account in [19,27], and a fixed UAV altitude assumption is considered in [5]. It is well known that the altitude of the UAV plays a major role in the performance of the network, due to its impact on the path loss, coverage radius, and the probability of line of sight (PLoS). In our work, we have considered a feasible altitude range to exploit the gain from altitude diversity.

The novelty and the key contributions of this paper can be summarized as follows.

- A multi-UAV and multi-UE system, where UEs are randomly distributed in a disaster struck area is considered.
- Algorithms are proposed to position the UAV ABSs and allocate UEs for each ABS, to maximize the sum spectral efficiency of the network, while maintaining a minimum QoS level for all UEs.
- The proposed scheme is centralized and has a low level of complexity, as only the statistical CSI, locations of the UEs, and the initialized locations of the ABSs are required as inputs.
- The proposed scheme allows the ABSs to directly move from their initial position to the optimal position with a single maneuver, making it a quick and energy efficient approach.
- The available energy levels in the batteries of the ABSs are taken into consideration in the deployment.

The remainder of this paper is organized as follows. In Section 2, we present our system model. Section 3 describes the proposed ABS placement and UE association schemes. Section 4 presents numerical results and insights, while Section 5 concludes this paper.

## 2. System Model

### 2.1. Spatial Model

**Figure 1:**
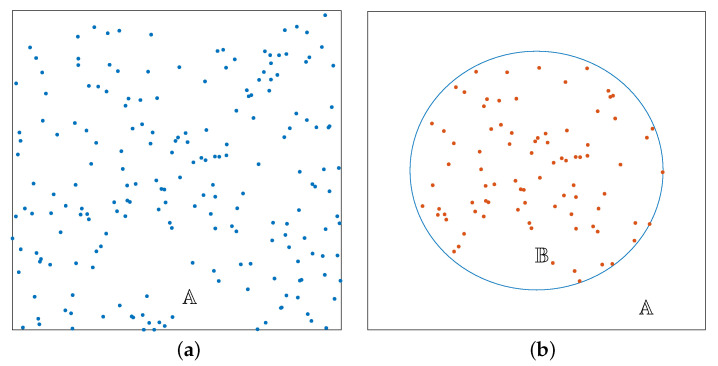
(**a**) User equipment (UE) distribution in A and (**b**) UE distribution in B (disaster region).

Consider an area A where the UEs are distributed following a homogeneous Poisson point process (PPP) with intensity of $\lambda_{U}$ in the 2-dimensional Euclidean space $R^{2}$. Due to a disaster, the UEs located inside the circular region of radius $R_{B}$ denoted by B (centered at the origin of A) have lost connectivity with the terrestrial network. Figure 1 illustrates a sample UE distribution. Hereafter, we only focus on the UEs in region B. The average number of UEs in B is $N_{\mathrm{UE}}$. To serve these UEs, $N_{\mathrm{UAV}}$ UAVs are deployed as ABSs. It is assumed that the maximum number of UEs supported by an ABS is $N_{T}$. Therefore, on the average, we have

(1) $$N_{\mathrm{UAV}}=\lceil \frac{N_{\mathrm{UE}}}{N_{T}}\rceil \;,$$

where the operator ⌈.⌉ represents the ceiling function.

Initially, the ABSs are deployed randomly in the 3-dimensional space above B. Let $S_{j}$ denote the coordinates of the jth ABS. $S_{j}$ takes the form of ($x_{j},y_{j},H_{j}$), where $x_{j}$ and $y_{j}$ represent the location of the jth ABS in $R^{2}$ and $H_{j}$ represents the altitude of the jth ABS. We define $S=\{S_{1},\ldots ,S_{N_{\mathrm{UAV}}}\}$. $B_{i}$ represents the location of the ith UE. Let $\phi_{j}$ denote the set of UEs associated with the jth ABS. We define the set $\Phi =\{\phi_{1},\ldots ,\phi_{N_{\mathrm{UAV}}}\}$, such that the sum of the cardinalities of $\phi_{j}$, $j\in \{1,2,\ldots ,N_{\mathrm{UAV}}\}$ is $N_{\mathrm{UE}}$, i.e., $\sum_{j}\left|\phi_{j}\right|=N_{\mathrm{UE}}$. Moreover we assume that a UE cannot communicate with more than one ABS, thus the elements in Φ are disjoint, i.e., $\phi_{j}\cap \phi_{i}=Ø$ for $\forall i\neq j\in \{1,\ldots ,N_{\mathrm{UAV}}\}$.

The ABSs have limited battery resources, and it is assumed that they cannot be recharged while in operation. For simplicity, we consider equal initial battery life at all ABSs. The available energy in batteries is used for both ABS maneuvering and data transmission. The total energy available for maneuvering is denoted by $E_{T}$. Although having equal battery power at initialization, the battery levels among ABSs shall differ while in operation, as per the distance traveled. Thus, each ABS keeps track of the available energy in the battery. The energy remaining for the maneuvering of the jth ABS is denoted by $E_{j}$. Considering a single maneuver of the jth ABS, the energy required to move the ABS from the present location ($a_{j}^{0},b_{j}^{0},c_{j}^{0}$) to the new location ($a_{j},b_{j},c_{j}$) is estimated using

(2) $$E_{\mathrm{mob}}^{j}=\eta_{h}{\left[{\left(a_{j}^{0}-a_{j}\right)}^{2}+{\left(b_{j}^{0}-b_{j}\right)}^{2}\right]}^{\;1/2}+\eta_{v}{\left[c_{j}^{0}-c_{j}\right)}^{2}{]}^{\;1/2},$$

where $\eta_{h}$ and $\eta_{v}$ denote the energy consumption for movement per unit distance in the horizontal and vertical directions, respectively.

### 2.2. Channel Model

As UEs are only served by ABSs, we only focus on the ABS-UE channel. This channel experiences both LoS and NLoS propagation conditions depending on the altitude of the ABSs. Therefore, it is essential to consider both LoS and NLoS links in a realistic performance evaluation. The probability of communicating through a LoS ABS-UE link can be calculated based on the elevation angle of an ABS with respect to a UE using the following originally given in [31],

(3) $$P\left(LOS,\theta_{j}^{i}\right)=\frac{1}{1+a\exp(-b\left[\theta_{j}^{i}-a\right])}\;\;,$$

where $\theta_{j}^{i}$ is the elevation angle of the jth ABS with respect to the ith UE, and *a* and *b* are environment dependent parameters.

By considering the effects of LoS and NLoS propagation, the channel gain from the jth ABS to the ith UE is modeled as

(4) $$h_{q}\left(j,i\right)=\frac{|g_{q}{|}^{2}}{\sqrt{{\left(H_{j}^{2}+d{\left(j,i\right)}^{2}\right)}^{\alpha_{q}}}},$$

where q∈{L,N} such that *L* and *N* refer to the LoS and NLoS conditions, respectively, $g_{q}$ is the small-scale fading amplitude, d(j,i) is the distance between the jth ABS and the ith UE, $\alpha_{q}$ is the large-scale path loss exponent [32], and we assume $\alpha_{L}<\alpha_{N}$. It is also assumed that $g_{L}$ follows a Rician fading distribution with the Rice factor *K*, while $g_{N}$ follows the Rayleigh fading distribution.

### 2.3. Signal-to-Interference-plus-Noise Ratio (SINR)

**Figure 2:**
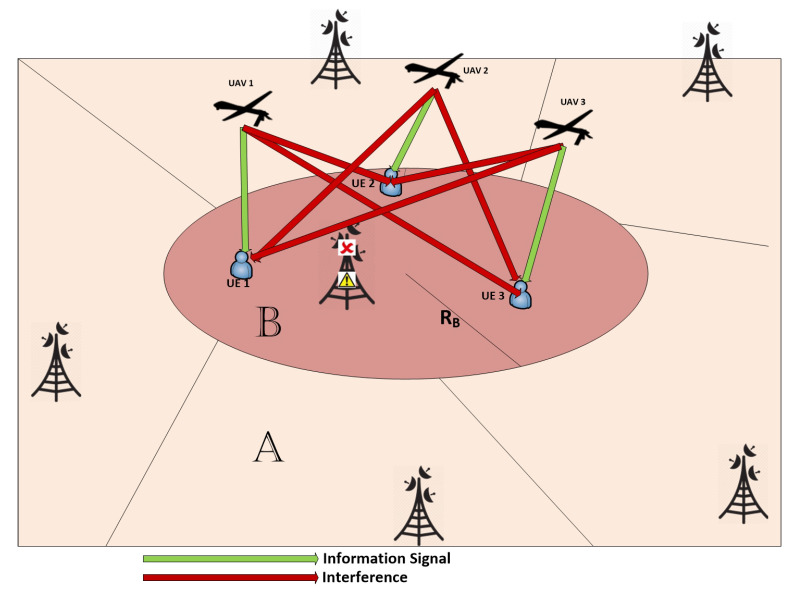
System model illustration of the information and interference signals for $N_{\mathrm{UAV}}=3$ and $N_{\mathrm{UE}}=3$.

Considering the interference from other co-channel ABSs is crucial when positioning an ABS [3]. In contrast to the works in [3,9,26], we consider the full impact of CCI in the ABS placement and UE association problems. Figure 2 illustrates a sample scenario of our proposed system. The SINR of the ith UE associated with the jth ABS is given by

(5) $$SINR\left(j,i\right)=\frac{P_{r}\left(j,i\right)}{I_{\mathrm{Agg}}\left(j\right)+N_{0}}\;\;,$$

where $P_{r}\left(j,i\right)$ is the signal power received at the ith UE from the jth ABS, $I_{\mathrm{Agg}}\left(i\right)$ is the aggregate interference experienced by the ith UE, and $N_{0}$ is the power spectral density of the Gaussian noise. In addition, the received signal power $P_{r}\left(j,i\right)$ can be expressed as

(6) $$P_{r}\left(j,i\right)=p_{t}^{j}\left[P\left(LOS,\theta_{j}^{i}\right)h_{L}\left(j,i\right)+\left(1-P\left(LOS,\theta_{j}^{i}\right)\right)h_{N}\left(j,i\right)\right]\;\;,$$

where $p_{t}^{j}$ is the transmit power of the jth ABS. The aggregate CCI is given by

(7) $$I_{\mathrm{Agg}}\left(i\right)=\sum_{l=1,l\neq j}^{N_{\mathrm{UAV}}}p_{t}^{l}\left[P\left(LOS,\theta_{j}^{i}\right)h_{L}\left(j,i\right)+\left(1-P\left(LOS,\theta_{j}^{i}\right)\right)h_{N}\left(j,i\right)\right],$$

where we have incorporated the effect of both LoS and NLoS propagation from the interfering ABSs.

## 3. Optimal ABS Placement and User Association

In this section, we focus on inferring S, the position of each ABS, and Φ, the assigned UE list for each ABS such that the total spectral efficiency (TSE) of the network is maximized. The TSE is given by

(8) $$TSE=\sum_{j=1}^{N_{\mathrm{UAV}}}\sum_{i\in \phi_{j}}^{}\log_{2}\left[1+SINR(j,i)\right].$$

The optimization problem of interest can be formulated as follows:

(9) $$\begin{array}{cc} \max_{S,\Phi }\; & TSE\\ s.t.\; & E_{\mathrm{mob}}^{j}\leq E_{j},\\ \; & SINR\left(j,i\right)\geq SINR_{\min},\;i\in \phi_{j},\;j\in \left\{1,\ldots ,N_{\mathrm{UAV}}\right\},\\ \; & \sum_{j=1}^{N_{\mathrm{UAV}}}\left|\phi_{j}\right|=N_{\mathrm{UE}},\\ \; & \phi_{j}\cap \phi_{i}=Ø,\;\forall i\neq j\in \left\{1,\ldots ,N_{\mathrm{UAV}}\right\},\\ \; & \left|\phi_{j}\right|\leq N_{T}\;j\in \left\{1,\ldots ,N_{\mathrm{UAV}}\right\}, \end{array}$$

where $SINR_{\min}$ is the SINR threshold set to ensure the minimum QoS requirement.

**Figure 3:**
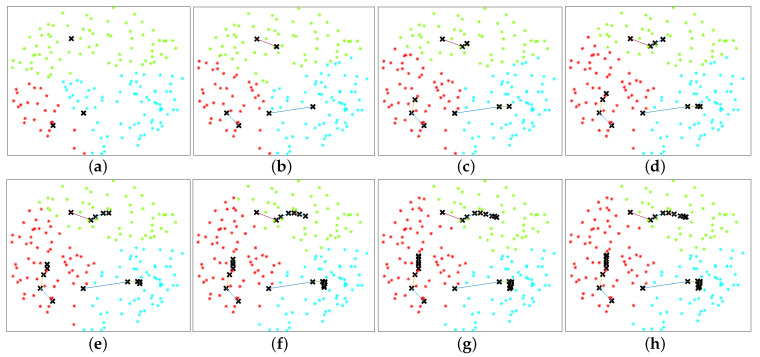
Illustration of ABS placement and UE association obtained using the approach in [14], where $R_{B}$ = 2000 m, $\alpha_{N}=2.5$, $\alpha_{L}=2$, $\lambda_{U}=2\times 10^{-4}$/m${}^{2}$, δ=0, $N_{\mathrm{UAV}}=3$, $H^{*}$= 300 m, $N_{T}=70$. The position of the ABS is represented using **X**. The three colors differentiate the UE clusters at a particular stage. (**a**–**h**) illustrate the 1st, *…*, 5th, 7th, 9th and 11th adaptive stages, respectively

It is clear that the objective function and the constraints of Equation (9) are non-convex and it is challenging to obtain an optimal solution with polynomial complexity. A problem very similar to Equation (9) has been solved in [14] using game theory combined with an iterative algorithm. Figure 3 illustrates an example ABS placement and UE association obtained using the approach in [14]. To reach this solution, in [14], it has been assumed that the small-scale fading process is stationary and the UEs and the ABSs have full knowledge of the CSI. However, it is important to note that the time it takes to move an ABS can be significantly larger than the coherence time of the channel, leading to decisions based on outdated CSI. Furthermore, it is important to note that the ABSs have to execute multiple maneuvers to reach the optimal 2D position, resulting in high energy consumption. Therefore, we consider a novel approach where only the statistical CSI and the locations of the UEs and the ABSs are used to determine the optimal 2D position and the UE assignment. In addition, we use a centralized approach to solve the placement and UE association problem, and move the ABSs to their optimal positions using a single maneuver to reduce the overall energy consumption.

To this end, this work defines a new SINR parameter based on statistical CSI and we refer to it as the *statistical SINR* (SSINR). The SSINR of the ith UE associated with the jth ABS is given by

(10) $$SSINR\left(j,i\right)=\frac{E[P_{r}\left(j,i\right)]}{E\left[I_{\mathrm{Agg}}\left(j\right)\right]+N_{0}}\;\;,$$

where E[·] denotes the expectation. From Equations (4), (6), and (7), it can be identified that SSINR(j,i) can be computed using the knowledge of UE and ABS positions and the statistics of Rayleigh and Rician fading. Therefore, compared to instantaneous CSI, a significantly lower overhead is required to make these information available at the CC. Using SSINR, we reformulate the optimization problem as

(11) $$\begin{array}{cc} \max_{S,\Phi }\; & STSE\\ s.t.\; & SSINR\left(j,i\right)\geq SINR_{\min},\;i\in \phi_{j},\;j\in \left\{1,\ldots ,N_{\mathrm{UAV}}\right\}, \end{array}$$

where the constraints not shown remain unchanged from Equation (9), and STSE is defined as

(12) $$STSE=\sum_{j=1}^{N_{\mathrm{UAV}}}\sum_{i\in \phi_{j}}^{}\log_{2}\left[1+SSINR(j,i)\right],$$

which can be interpreted as the achievable TSE when SINR(j,i) is approximated by SSINR(j,i), and we refer to STSE as *statistical total spectral efficiency*. It is not hard to recognize that Equation (11) is also non-convex and cannot be solved using algorithms with polynomial complexity. Therefore, we propose to solve Equation (11) using a 3-step approach, namely, the 2D deployment of the ABSs, UE assignment, and altitude selection of the ABSs. This paper first presents a methodology for the 2D deployment of the ABSs and the UE assignment.

### 3.1. 2D Deployment of the ABSs and the UE Assignment

**Figure 4:**
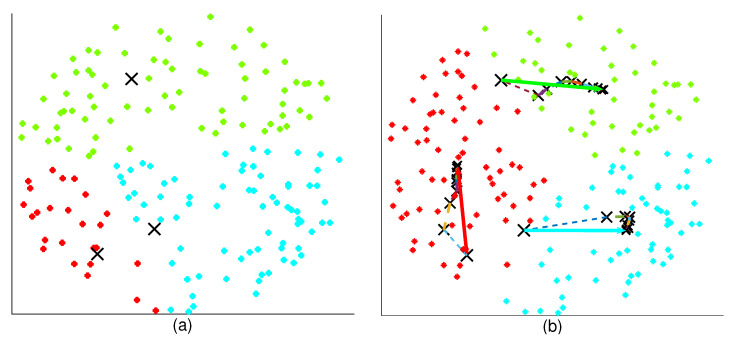
Illustration of the movement of the aerial base stations (ABSs) in the 2D plane for suburban environment. The position of the ABS is represented using **x**. The three colors differentiate the UE clusters of the respective ABSs. (**a**) Initial 2D position of the ABSS. (**b**) Movement of the ABSs to the computed position. The solid arrow represents the actual ABS movement. The doted lines represent the adaptive process (does not represent the movement) performed at the CC. $R_{B}$ = 2000 m, $\alpha_{N}=2.5$, $\alpha_{L}=2$, $\lambda_{U}$ = 2 × 10${}^{-4}$/m${}^{2}$, δ=0, $N_{\mathrm{UAV}}=3$, $H^{*}$ = 300 m, $N_{T}=70$.

Initially, all ABSs are randomly and uniformly placed above the disaster zone. The ABSs then estimate the locations of the UEs in its region of coverage from the uplink signals. These locations are sent to a centralized location, together with the locations of the ABSs, such that the centralized location is fully aware of the network topology. Using this knowledge, the user assignment and the 2D positioning follows an iterative three step process. Note that all of these iterations take place at the centralized location. First, the received SSINR values at each UE from the ABSs providing coverage are calculated. Second, using the calculated SSINRs, the UE assignment to the ABSs is performed through a stable marriage approach. The goal is to assign the UEs to the ABS providing the best SSINR such that the constraint on the maximum UEs per ABS is not violated. Therefore, in the stable marriage approach, the UE assignment is done such that both these parties (UEs and ABSs) are jointly satisfied with a particular assignment, and there is no other UE assignment that the two parties would rather prefer having than the current assignment. If there are no such other assignments, the matches are deemed stable. Once each UE is assigned to an ABS, the third step of the 2D deployment of the ABSs is done through a clustering algorithm. The clustering algorithm proposed in this paper closely follows the principles of K-means clustering. However, in contrast to conventional K-means clustering, our algorithm uses multiple weighting parameters in the decision phase. Essentially, the locations of the ABSs are updated such that they coincide with the centroid of the locations of their assigned UEs in the 2-dimensional Euclidean space, denoted by $\left(x_{c},y_{c},0\right)$. This three step process continues until the exit condition is satisfied. The statistical total spectral efficiency gain denoted by $G_{L}$ is used as the metric to determine the algorithm convergence. To this end,

(13) $$G_{L}=\psi^{n}-\psi^{n-1}\;,$$

where $\psi^{n}$ is the STSE in the nth iteration. The iterative process continues as long as $G_{L}$ is greater than δ, which is the least expected gain from an iteration. Note that the physical locations of the ABSs do not change iteratively, thus they can hover at the initialized location until a decision is made on the optimal location. Figure 4 illustrates the movement of the ABSs in the 2D plane after finding the best 2D position through CC.

### 3.2. ABS Altitude Selection

We present two approaches for the altitude selection. In the first approach, the altitude diversity is not considered and all ABSs are assumed to be at the same optimal altitude, denoted by $H^{*}$. Once the initial optimal 2D positions are determined, an exhaustive search over a discrete set of altitudes is used to find the optimal altitude for the ABSs. For each altitude, the 2D locations and UE assignments are further fine-tuned, using the same three-step iterative process described earlier. To limit the search space in the exhaustive search, we make the following observations regarding the received SSINR of a UE. Considering the impact of the ABS altitude in the downlink, it is shown in [31] that when CCI is not present, the received signal power increases in the beginning and decreases after a certain point, indicating the existence of an optimal altitude where the received power is maximum. This behavior can be explained using the combined effect of path loss and the probability of line of sight. The path loss increases with the ABS altitude, resulting in the degradation of the signal quality. However, increasing the altitude also leads to higher P(LOS,θ), which in turn results in better signal quality. Once the altitude increases beyond a critical point, the improvement in P(LOS,θ) becomes negligible compared to the signal power degradation due to path loss. Therefore, the received signal power increases in the beginning and decreases after a certain point, when the altitude of the ABSs hovering at ground level is increased. However, the reasoning is slightly different when CCI is considered. For a typical UE, the interfering ABSs have larger link lengths compared to the connected ABS, when the ABSs are at lower altitudes. Therefore, the interference power is smaller compared to the desired signal power at lower altitudes. Thus, the SSINR will behave similarly to the case with no CCI. In higher altitudes, the SSINR degrades due to two factors. First, the path loss increases and starts to dominate the desired signal power compared to the effect from P(LOS,θ). Second, the increased elevation angles with the interfering ABSs further increase the interference power. Therefore, the SSINR decreases continuously after a critical altitude. This means the STSE increases with the common altitude of the ABSs up to a certain altitude level, and then decreases monotonically. This intuition is used to limit the search space of the exhaustive search, as there is no advantage searching if the objective function is in the decreasing trend. Therefore, in the first approach, the common ABS altitude is increased until the STSE begins to decrease, and an optimal altitude is found accordingly. These ideas are formally stated in Algorithm 1. Notations used in the algorithms are tabulated in Table 1.

The second approach, which we present as Algorithm 2, resorts to the same approach for 2D positioning of the ABSs and for user association as in Algorithm 1. However, Algorithm 2 uses particle swarm optimization (PSO) to set the altitude values of the ABSs. The approach facilitates altitude diversity. PSO is an intelligent algorithm that stems on the approach used by a group of birds for searching food or for traveling long distances. Essentially, this algorithm keeps track on two positions, namely the local best (LB) and the global best (GB). $W_{k}^{\mathrm{Lb}}\left(n\right)$ denotes the LB of the kth particle in the nth iteration, and $W^{\mathrm{Gb}}\left(n\right)$ is the GB in the nth iteration. The LB gives us the optimal solution (position) for a particular particle where as the GB gives us the optimal solution (position) among all the particles. The velocity vector of a particle is calculated based on the LB, the GB and the inertia of the particle. To this end, the velocity of the kth particle during the nth iteration is given by

(14) $$V_{k}\left(n\right)=\xi V_{k}\left(n-1\right)+c_{1}\varphi_{1}\left(W_{k}^{\mathrm{Lb}}\left(n-1\right)-W_{k}\left(n-1\right)\right)+c_{2}\varphi_{2}\left(W_{k}^{\mathrm{Gb}}\left(n-1\right)-W_{k}\left(n-1\right)\right),$$

where ξ is the inertia weight that controls the exploration ability, $\varphi_{1}$ and $\varphi_{2}$ are positive random numbers, and $c_{1}$ and $c_{2}$ denote the local learning coefficient and the swarm learning coefficient, respectively. Moreover, the position of the kth particle in the nth iteration is updated according to

(15) $$W_{k}\left(n\right)=W_{k}\left(n-1\right)+V_{k}\left(n\right).$$

**Figure 5:**
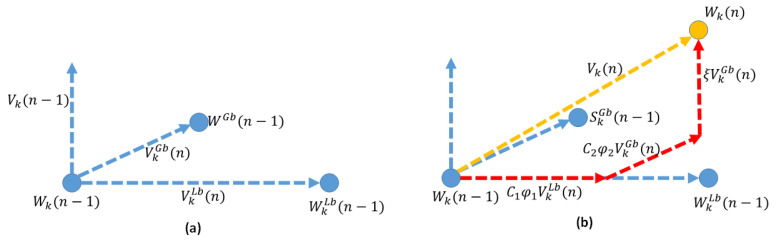
(**a**) Global best, local best, position, and the velocity in the (n−1)th iteration. (**b**) Velocity in the nth iteration as a weighted vector addition of previous velocity components and the position in the nth iteration.

The movement of the kth particle in the PSO problem space is illustrated in Figure 5. The next position of each particle in the PSO space will be decided upon three vectors, which are the GB vector, the LB vector, and the previous velocity vector.
**Algorithm 1:** Clustering and matching algorithm with exhaustive search. 
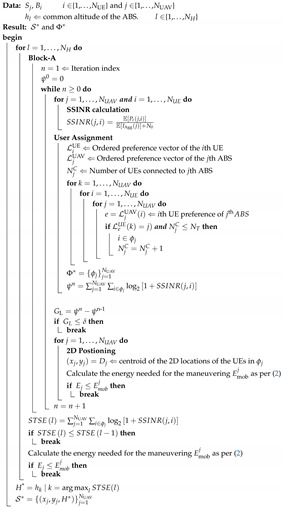


With regards to our problem, Algorithm 2 initializes the PSO population with their positions. Each particle is a vector of size of $N_{\mathrm{UAV}}$, such that its jth element represents the altitude of the jth ABS. Initially, the altitude values in all particles, i.e., each element in the vector, are set randomly and uniformly between the minimum and the maximum allowable altitudes. These values are used to initialize the LB of each particle, and the initial velocity of each particle is set to one. For each particle, i.e., for each altitude vector, the corresponding optimal UE assignment and the optimal 2D position allocation are obtained through **Block-A** of Algorithm 1. Then, the objective function, which is the STSE, is evaluated for each particle considering the current UE and the position assignments. Their initial position is the LB for all the particles and is also based on these calculated values; the GB position vector of the swarm will be updated. This ends the initialization stage.

Then, the iterative process of finding the best set of locations begins. The velocity and the altitude of each particle are updated as per (14) and (15), respectively. For each particle, i.e., for each altitude vector, the corresponding optimal UE assignment and the optimal 2D position allocation are obtained through **Block-A** of Algorithm 1. The objective function is computed again for the newly updated positions, and the LB and GB positions are updated consequently.

The iterative process continues until the STSE gain between two subsequent iterations, denoted by $G_{P}$, is below a certain predefined threshold. More precisely, if there are Γ subsequent iterations where the $G_{P}$ is less than a predetermined threshold value $\tilde{\delta }$, the iterative process stops and the PSO is assumed to have reached convergence. At convergence, GB contains the altitude vector for the ABSs that maximizes the TSE as per Algorithm 2. These ideas are formally stated in Algorithm 2.
**Algorithm 2:** Clustering and matching algorithm with PSO 
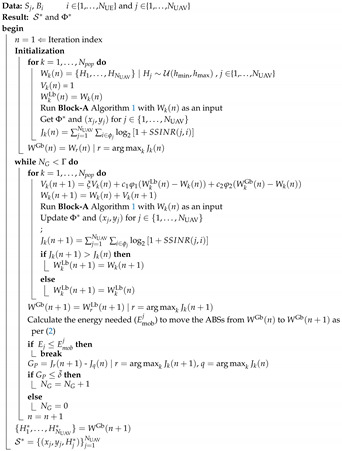


## 4. Simulation Results and Discussion

In this section, the performance of the proposed algorithms is evaluated through extensive simulation results. The parameters used in the simulations are given in Table 2. The algorithms are compared in terms of TSE, energy consumption, and the average coverage probability, which is computed as

(16) $$\bar{P_{\mathrm{COV}}}=\frac{N_{\gamma_{\mathrm{th}}}}{N_{\mathrm{UE}}}\;\;,$$

where $N_{\gamma_{\mathrm{th}}}$ is the number of users experiencing SINR greater than a threshold value $SINR_{\min}$. In simulations, we consider A to be a $6\times 6\;{\mathrm{km}}^{2}$ square. To be compatible with the literature, other parameters are set as in Table 2. Moreover, the gain thresholds, δ and $\tilde{\delta }$, are set to 0 as we assume static UEs.

**Figure 6:**
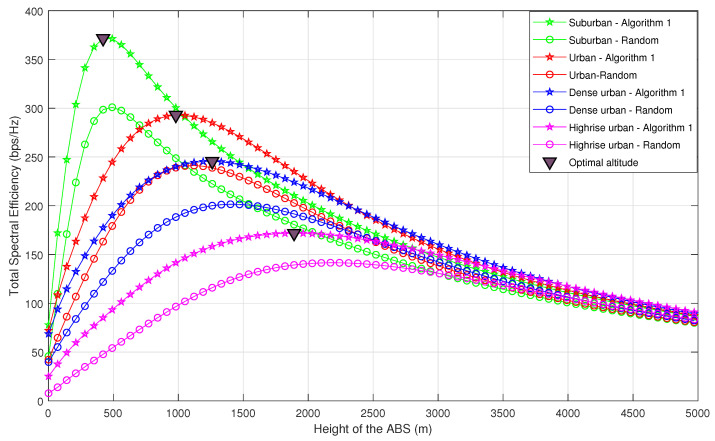
Total spectral efficiency vs. altitude of the ABS (comparison between Algorithm 1-based deployment and random deployment).

**Figure 7:**
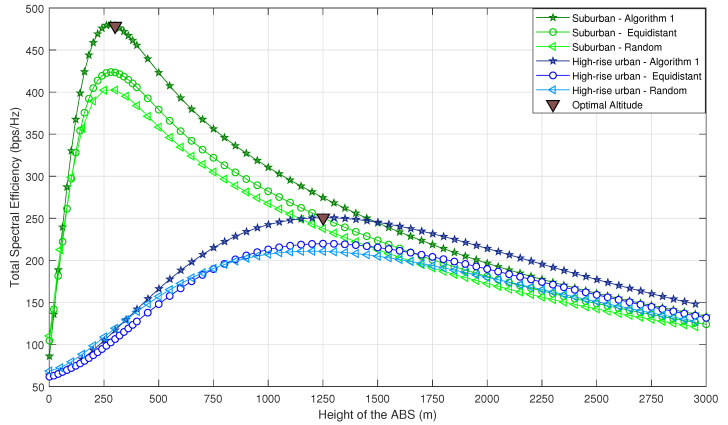
Total spectral efficiency vs. altitude of the ABS (comparing Algorithm 1-based deployment, random deployment, and equidistant deployment).

Figure 6 and Figure 7 compare the achievable TSE of Algorithm 1 for different ABS altitudes, with naive random ABS deployment and the equidistant deployment. In naive random deployment, the 2D locations of the ABSs above B are chosen randomly. The UEs are assigned to the nearest ABS such that each UE is assigned to only one ABS, in a manner that all UEs meet the minimum QoS requirement ($SINR_{\min}$). In equidistant deployment, the ABSs are placed along radial lines that equally partition a circle above B. The ABSs move outwards or inwards radially, until all the UEs meet the minimum QoS requirement $SINR_{\min}$. It can be observed that Algorithm 1 outperforms the random and equidistant deployments. Furthermore, one can note that even though we have used SSINR and STSE in obtaining the solution, the optimal altitude obtained by our algorithm is actually the altitude which provides the maximum TSE for the considered propagation environments.

Moreover, the optimal altitudes for the considered propagation environments in the ascending order are suburban, urban, dense urban, and high-rise urban. As explained in Section 3, the impact of P(LOS,θ) on the signal quality is dominant until it starts to saturate. It is important to note that the elevation angles at which P(LOS,θ) begins to saturate also follow the same order. Therefore, the optimal altitude is smallest for suburban environments while it is largest for high-rise urban environments. This is a valuable insight when designing ABSs for different environments, as ABSs must be designed with sufficient energy to reach the optimal altitude. Furthermore, it can be observed that TSE in all scenarios converge to the same value for higher ABS altitudes regardless of the propagation environment. This is because for higher altitudes, regardless of the propagation environment, θ approaches 90${}^{\circ }$, leading to P(LOS,θ)≈1. Therefore, TSE solely depends on path loss, which is almost the same in all environments.

**Figure 8:**
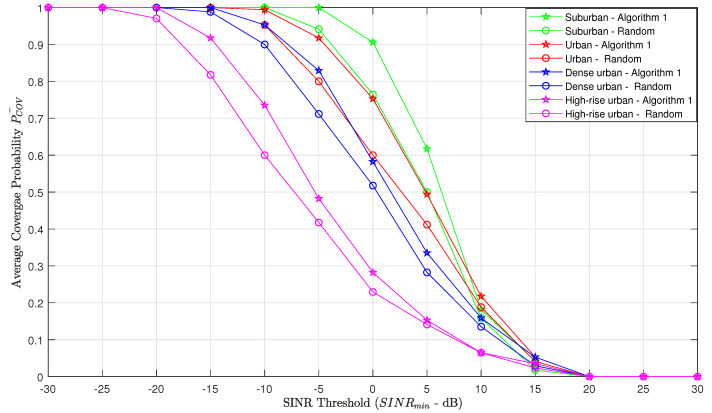
Average coverage probability vs. Signal-to-Interference-Plus-Noise Ratio (SINR) threshold (comparison between Algorithm 1-based deployment and random deployment).

To compare Algorithm 1 with naive random deployment in terms of coverage probability, we evaluate the average coverage probability for each propagation environment, when the ABSs are placed at their optimal altitudes. From Figure 6, the optimal altitudes of the ABSs are considered as 300 m, 650 m, 800 m and 1250 m for suburban, urban, dense urban and high-rise urban, respectively. Figure 8 shows how the coverage probability behaves with the SINR threshold. It is conspicuous that the average coverage probability of Algorithm 1 is superior to random deployment.

**Figure 9:**
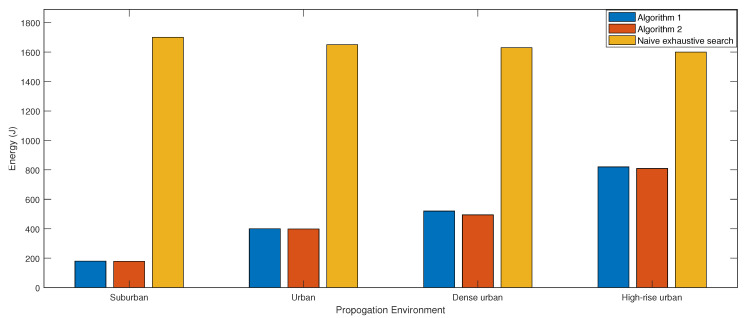
Energy consumption of Algorithms 1 and 2 compared to naive exhaustive search.

Figure 9 presents the total consumed energy for ABS movements using Algorithm 1, Algorithm 2, and naive exhaustive search, where the search is performed over all possible altitudes. Both proposed algorithms have almost similar energy consumption, while being significantly lower (9-fold saving) than the naive exhaustive search. This is due to the reduced number of ABS movements we need to perform with the proposed centralized approach. It is interesting to note that energy saving decreases from suburban to high rise urban due to the increase in the optimal altitude.

**Figure 10:**
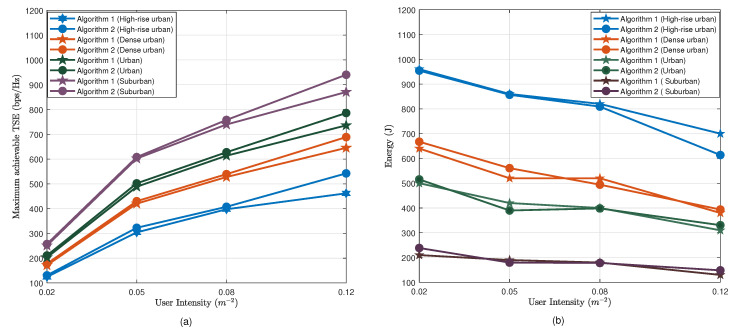
(**a**) Maximum achievable total spectral efficiency (TSE) vs. user intensity (**b**). Energy consumption for maneuvering vs. user intensity.

Figure 10 compares Algorithms 1 and 2 in terms of the maximum achievable TSE and the total consumed energy for ABS movements with different user densities in four propagation environments. One can observe that for dense networks, Algorithm 2 results in higher TSE compared to Algorithm 1. This clearly shows the advantage of altitude diversity for dense networks. Almost equal energy consumption can be observed for both algorithms. Furthermore, it can be observed that the total energy consumption decreases with UE density. It is clear that ABSs have to move only a small distance from the initial position to the optimal position, as in dense UE scenarios, the optimal 2D placement can be closely approximated by the uniform placement.

## 5. Conclusions

This paper investigated the problem of ABS placement and UE assignment to maximize the sum spectral efficiency of a disaster affected wireless network. K-means clustering combined with a stable marriage problem has been used to determine the 2D placement of the ABSs and the UE assignment, while two approaches based on exhaustive search and particle swarm optimization have been proposed to determine the optimal ABS altitude. The proposed algorithms use only the statistical channel state information and the location information of the UEs and ABSs. Useful design insights such as the optimal ABS altitude, total required energy for ABS movement have been presented for different propagation environments. The proposed algorithms result in up to 8-fold saving in the energy required to maneuver the ABSs, compared to ABS deployment using naive exhaustive search.

## Figures and Tables

**Table 1:** Table of notations.

Notation	Description
$x_{j},y_{j}$	2D- Coordinates of the jth ABS
$B_{i}$	2D- Coordinates of the ith UE
$\lambda_{U}$	Intensity of the UE distribution
$R_{B}$	Radius of the isolated region
$N_{\mathrm{UAV}}$	Required Number of UAVs
$N_{\mathrm{UE}}$	Number of UEs in the isolated region
$N_{T}$	Maximum number of UE that can be supported by an ABS
d(j,i)	2D euclidean distance from jth ABS to ith UE
$\alpha_{q}$	Large-scale path loss exponent
$g_{q}$	Small-scale fading amplitude
$p_{t}^{j}$	Transmission power of the jth ABS
$P_{r}\left(j,i\right)$	Received signal power at ith UE from the jth ABS
$I_{\mathrm{Agg}}\left(i\right)$	Aggregated interference experienced by the ith UE
$E_{\mathrm{mob}}^{j}$	Required energy for mobility of the jth ABS
$E_{j}$	Available energy for mobility at the jth ABS
$\eta_{h}$, $\eta_{v}$	Energy consumption per unit distance to horizontal and vertical movement respectively
$\phi_{j}$	Assigned user list of the jth ABS
$P(LOS,\theta_{j}^{i})$	probability of line of sight from jth ABS to the ith UE
a,b	Constants which reflects environmental characteristics
$h_{q}\left(j,i\right)$	Channel gain from the jth ABS to the ith UE
$SINR_{\min}$	Minimum SINR threshold which reflects the minimum QoS requirement
$G_{L}$	Gain achieved comparing to the previous step
$H_{j}$	Altitude of the jth ABS
$H^{*}$	Common optimal altitude
$N_{H}$	Number of discrete altitude levels considered in Algorithm 1
$H_{j}^{*}$	Optimal altitude of the jth ABS
δ, $\tilde{\delta }$	Minimum gain expected in Algorithm 1 and Algorithm 2
$h_{\min}$,$h_{\max}$	Minimum and maximum altitude allowed to hover an ABS
$W_{k}\left(n\right)$	Position of the $k^{\mathrm{th}}$ particle at nth iteration in PSO space
$W^{\mathrm{Gb}}\left(n\right)$	Global best position at the nth iteration in PSO space
$W_{k}^{\mathrm{Lb}}\left(n\right)$	Local best position of the $k^{\mathrm{th}}$ particle at nth iteration in PSO space
$V_{k}\left(n\right)$	Velocity of kth particle at nth iteration in PSO space
$J_{k}\left(n\right)$	Objective function value of the kth particle at nth iteration in PSO space
$c_{1},\;c_{2}$	Local learning coefficient and swarm learning coefficient respectively
ξ	Inertia weight of the swarm particle
$N_{\mathrm{pop}}$	Number of particles in the swarm population
$G_{P}$	Spectral efficiency gain achieved comparing to the previous iteration in PSO
$N_{G}$	Number of continuous iterations without a gain in the spectral efficiency
Γ	Threshold to exit the PSO algorithm

**Table 2:** Simulation settings.

Parameter	Value	Parameter	Value	Parameter	Value
$\lambda_{u}$	$4\times 10^{-4}$/m${}^{2}$	$R_{B}$	2000 m	*r*	15 Mbps
$N_{T}$	40	$E_{T}$	1 kJ	$\eta_{h}$	0.1 J/m
$\alpha_{L}$	2	$\alpha_{N}$	2.5	$\eta_{v}$	1 J/m
$p_{t}^{j}$	30 dBm	δ,$\tilde{\delta }$	0	$N_{\mathrm{pop}}$	20
Γ	4	$N_{0}$	−80 dBm	$\varphi_{1},\varphi_{2}$	random in [0, 1]
*a*	4.8800 (Suburban)	*b*	0.4290 (Suburban)	$SINR_{\min}$	−30 dB
	9.6117 (Urban)		0.1581 (Urban)	$h_{\min}$	50 m
	12.0810 (Dense urban)		0.1140 (Dense urban)	$h_{\max}$	3000 m
	24.5960 (High-rise urban)		0.1248 (High-rise urban)	ξ	0.5175
